# Scale-up of a novel vital signs alert device to improve maternity care in Sierra Leone: a mixed methods evaluation of adoption

**DOI:** 10.1186/s12978-022-01551-2

**Published:** 2023-01-06

**Authors:** Sophie Bright, Francis Moses, Alex Ridout, Betty Sam, Mariama Momoh, Venetia Goodhart, Francis Smart, Margaret Mannah, Sattu Issa, Simren Herm-Singh, Fiona Reid, Paul T. Seed, James Bunn, Andrew Shennan, Katrin Augustin, Jane Sandall

**Affiliations:** 1 School of Health and Related Research, University of Sheffield, Sheffield, England; 2 Reproductive Health and Family Planning, MoHS, Freetown, Sierra Leone; 3 King’s College London (KCL), London, England; 4 Welbodi Partnership, Freetown, Sierra Leone; 5 Ministry of Health and Sanitation (MoHS), Freetown, Sierra Leone; 6 Planning and Information, MoHS, Freetown, Sierra Leone; 7 Quality Management Programme, MoHS, Freetown, Sierra Leone; 8 Department of Population Health Sciences, KCL, London, England; 9 Department of Women and Children’s Health, KCL, London, England; 10 World Health Organization, Freetown, Sierra Leone; 11 School of Population Health and Environmental Sciences, KCL, London, England; 12 Women’s Health Academic Centre, KCL, London, England

## Abstract

**Background:**

The CRADLE (Community blood pressure monitoring in Rural Africa: Detection of underLying pre-Eclampsia) Vital Signs Alert device—designed specifically to improve maternity care in low resource settings—had varying impact when trialled in different countries. To better understand the contextual factors that may contribute to this variation, this study retrospectively evaluated the adoption of CRADLE, during scale-up in Sierra Leone.

**Methods:**

This was a mixed methods study. A quantitative indicator of adoption (the proportion of facilities trained per district) was calculated from existing training records, then focus groups were held with ‘CRADLE Champions’ in each district (n = 32), to explore adoption qualitatively. Template Analysis was used to deductively interpret qualitative data, guided by the NASSS (non-adoption, abandonment, scale-up, spread, sustainability) Framework.

**Findings:**

Substantial but non-significant variation was found in the proportion of facilities trained in each district (range 59–90%) [*X*^2^ (7, *N* = 8) = 10.419, *p* = *0.166*]. Qualitative data identified complexity in two NASSS domains that may have contributed to this variation: ‘the technology’ (for example, charging issues, difficulty interpreting device output and concerns about ongoing procurement) and ‘the organisation’ (for example, logistical barriers to implementing training, infighting and high staff turnover). Key strategies mentioned to mitigate against these issues included: transparent communication at all levels; encouraging localised adaptations during implementation (including the involvement of community leaders); and selecting Champions with strong soft skills (particularly conflict resolution and problem solving).

**Conclusions:**

Complexity related to the technology and the organisational context were found to influence the adoption of CRADLE in Sierra Leone, with substantial inter-district variation. These findings emphasise the importance of gaining an in-depth understanding of the specific system and context in which a new healthcare technology is being implemented. This study has implications for the ongoing scale-up of CRADLE, and for those implementing or evaluating other health technologies in similar contexts.

**Supplementary Information:**

The online version contains supplementary material available at 10.1186/s12978-022-01551-2.

## Plain language summary

Many women die during pregnancy and childbirth from causes that could be prevented, and the vast majority of these deaths occur in low-resource settings. The ‘CRADLE Vital Signs Alert’ is a medical device that helps identify problems during pregnancy—designed specifically for healthcare professionals in low-resource settings. However, for unknown reasons, the device appears to have varying impact according to the country or setting in which it is used. This study aimed to explore in depth whether, and why, healthcare professionals in Sierra Leone adopted the device and engaged in training (or not). Between March 2020 and January 2021, the CRADLE device and training package was disseminated across 8 districts in Sierra Leone. This relied on a few healthcare workers (nominated ‘CRADLE Champions’) to voluntarily distribute the devices and training in their local areas. Group discussions were held with CRADLE Champions in each district after the rollout to gather their feedback. In addition, the proportion of facilities trained in each district was recorded. The study found differences in how well the device and training was adopted in each district. Common challenges reported across districts related to technological difficulties (such as issues charging the devices) and organisational barriers (such as high levels of staff turnover at facilities). These findings will help to inform future rollout of the CRADLE device and training in Sierra Leone and highlight factors that may need to be considered by those implementing other health technologies in similar settings.

**Supplementary Information:**

The online version contains supplementary material available at 10.1186/s12978-022-01551-2.

## Background

### Maternal mortality in Sierra Leone

In 2020, the maternal mortality ratio (MMR) in Sierra Leone (SL) was estimated to be one of the highest in the world (1120 maternal deaths per 100,000 live births)—almost six times the global average [1]. Severe bleeding, infection and blood pressure disorders are the most common causes of maternal deaths, both in Sierra Leone, and globally [2, 3]. These complications are correlated with abnormal vital signs, which if detected early, can be managed with simple interventions. Accurately measuring vital signs is therefore critical [4]. However, in SL there is a dearth of critical monitoring equipment and senior healthcare staff, inhibiting timely identification and management of complications [5, 6]. There is therefore great capacity to benefit from improved vital signs monitoring in SL.

### The CRADLE vital signs alert

The CRADLE Vital Signs Alert (VSA) is a hand-held, upper-arm, semiautomated device, developed to accurately measure women’s vital signs throughout pregnancy and the postpartum period. The device measures blood pressure and heart rate, calculates shock index (pulse divided by systolic blood pressure), and digitally displays the results alongside a traffic light early warning system. A green light is displayed if the woman is at low risk of shock or high blood pressure, amber if she needs careful monitoring, or red if she requires emergency treatment. This early warning system is based on comparison of the calculated shock index against set thresholds that have been extensively validated for use in low-resource settings [7, 8]. An arrow pointing up or down is also displayed alongside a red or amber light, to indicate whether the issue is related to *high* or *low* blood pressure, further guiding the user on appropriate clinical management. The device was designed to be usable by any cadre of health-care provider, including those with little formal training (such as community healthcare workers) and has been specifically tailored for use in low-resource settings; it is low-cost, robust, portable, requires infrequent calibration and has low power requirements [9].

A previous qualitative evaluation of the CRADLE VSA found the device to be well accepted by both healthcare workers and service users, across a range of countries and healthcare settings. Between 2014 and 2016, 155 interviews and six focus groups were undertaken with healthcare workers, pregnant women, and their families, at sites trialling the device across India, Mozambique, Nigeria, and South Africa. Most healthcare workers reported that the device was easy to use and perceived it to be accurate, whilst pregnant women unanimously liked the device, reporting that it increased their understanding of vital signs monitoring [10].

Between 2016 and 2017, CRADLE was introduced across eight low- and middle- income countries, as part of a pragmatic, stepped-wedge, cluster-randomised controlled trial (CRCT) [11] . Post intervention, there was an 8% reduction in primary composite outcome (at least one of eclampsia, emergency hysterectomy and maternal death). However, due to substantial variability within and between clusters, no significant benefit could be attributed directly to the intervention. A concurrent mixed-methods process evaluation found considerable variation in the implementation, reach, adoption, and context between sites, which may have contributed to variation in impact [12], although no significant association was found between the composite ‘implementation strength score’ and the primary outcome (OR 0.93; 0.07–13.01).

### Purpose of this evaluation

Promising technological innovations in healthcare are often hindered by problems of non-adoption or abandonment by individuals and/or difficulties with scale-up and spread within organisations [13]. Provision of the CRADLE device and training package is therefore not a solution in itself, and consideration must be given to how well the device is adopted within the context of a specific healthcare system. Due to the variation in impact and process seen in the CRCT, further in-depth evaluation at individual sites was recommended [12]. Sierra Leone has been the first country to implement CRADLE at scale, providing a unique opportunity to evaluate the adoption of CRADLE outside of a clinical trial setting, where real-world contextual issues may be more apparent.

This retrospective evaluation of the CRADLE scale-up in SL is supported by the evidence-based NASSS (non-adoption, abandonment, scale-up, spread, sustainability) Framework—developed to help predict and evaluate the success of technology-supported health interventions [14]. The Framework consists of seven domains: the technology; the value proposition; the adopter system; the organisation; the wider system; and embedding and adaptation over time. If an intervention is found to have complexity in several domains (i.e. the domains are dynamic, unpredictable and/or are not easily disaggregated into constituent components) an intervention is considered less likely to be adopted and sustained. Guided by the NASSS framework, this evaluation aims to:

- Identify any inter-district variations in adoption of CRADLE in SL
- Explore the factors impacting upon adoption
- Provide guidance on how to avoid non-adoption and abandonment

## Methods and ethical considerations

### Context and intervention

Sierra Leone has a fragmented healthcare system, hindered by its colonial history, a long civil war (1991–2002) and the 2013 Ebola outbreak [15]. The quality of maternal health services is poor, with few women receiving expected standards of care [16]. The CRADLE device and training package was scaled-up in eight of SL’s sixteen districts, between March 2020 and January 2021, using a ‘training-of-trainers’ model [17]. National obstetric experts trained five Master Trainers, who together trained twenty CRADLE Champions (healthcare providers asked to incorporate training into their existing roles) per district. Champions were given materials and asked to disseminate training at all Ministry of Health and Sanitation (MoHS) facilities within a designated catchment area. Implementation districts were selected by the MoHS and included both urban and rural areas.

### Methods and analysis

The evaluation took a pragmatic, mixed methods approach—as recommended for the evaluation of health programmes [18].

#### Quantitative

An existing database of training registers, held by the implementing organisation, was used to calculate the proportion of facilities trained per district. This served as a proxy of adoption (by both the CRADLE Champions and the facilities offered training). This information was used to guide the selection of districts for the qualitative evaluation, aiming to choose a sample most representative of the spread of adoption levels. A chi-squared goodness-of-fit test was also performed to test whether inter-district differences in adoption were statistically significant.

#### Qualitative

Focus Group Discussions (FGDs) were held with Champions in the four selected districts, to explore their views and experiences of the CRADLE intervention and reflections on how it was adopted by others. Around half of the Champions in each district were invited to participate (total invited n = 41). Champions were purposefully selected to represent the range of Champion demographics. A topic guide was used flexibly and questions open-ended, to maximise breadth of responses and allow for unanticipated adoption-related topics. Each FGD had two moderators, one Sierra Leonean (BS) and one British (SB), both female. Discussions were in English,  but use of local languages was permitted where necessary to convey meaning. Discussions were audio-recorded, and field notes taken.

Qualitative data were interpreted via Template Analysis [19]. Analysis was primarily deductive, using the domains of the NASSS Framework as a priori themes. However, development of the coding Framework was an iterative process, remaining open to the refinement or removal of themes where appropriate. A subtle-realist stance underpinned the analysis i.e. the belief that the researcher’s analysis is influenced by their social stance, but that phenomena are to an extent knowable through the research process [20].

## Results

### Quantitative

The proportion of facilities trained varied from 59 to 90% per district (mean 77.5%; Table 1). This variation, although substantial, was not statistically significant [χ^2^ (7, N = 8) = 10.419, p = 0.166]. Four districts with varying adoption levels, including those with the highest (D, H) and lowest (A, B) adoption, were invited to participate in FGDs. The districts aren’t named to ensure participant confidentiality.

**Table 1:** Proportion of facilities trained disaggregated by district (selected FGD districts in bold)

District	Number of targeted facilities	Number of facilities trained	% trained
**District A**	**68**	**40**	**59**
**District B**	**81**	**50**	**50**
District C	65	47	72
**District D**	**71**	**57**	**80**
District E	142	119	84
District F	85	73	86
District G	126	109	87
**District H**	**103**	**93**	**90**

### Qualitative

FGDs (n = 4), lasted 109–141 min. The time between initial training and FGDs was 8–12 months, giving all districts adequate implementation time. Participation rate was 78%, with at least seven participants per district. Participant demographics are presented in Table 2. The majority were Female (94%), Midwives (78%), and based at Community Health Centres (53%).

**Table 2:** Demographics of focus group discussion participants

	District A	District B	District H	District D	Total	% of sample
Total	8	10	7	7	**32**	100
Cadre						
Midwife	8	10	6	1	25	78%
SRN	0	0	0	0	0	0%
SECHN	0	0	1	4	5	16%
CHO	0	0	0	1	1	3%
MCH Aid	0	0	0	1	1	3%
Gender						
Male	1	1	0	0	2	6%
Female	7	9	7	7	30	94%
Age (years)						
18–24	0	0	0	0	0	0%
25–34	0	2	0	3	5	16%
35–44	3	5	5	2	15	47%
45–54	4	2	2	2	10	31%
55–65	1	1	0	0	2	6%
Experience in healthcare (years)						
1–5	0	1	0	2	3	9%
6–10	0	2	1	4	7	22%
11–15	3	5	3	1	12	38%
> 15	5	2	3	0	10	31%
Place of work						
Hospital	1	1	2	1	5	16%
Community Health Centre	3	8	3	3	17	53%
Community Health Post	3	1	2	2	8	25%
Maternal and Child Health Post	0	0	0	1	1	3%
Other	1*	0	0	0	1	3%

*Maternal and Child Health Aide Training School

Using Template Analysis, all codes aligned with one of the seven NASSS domains. The two most dominant domains were ‘The Technology’ (152 references) and ‘The Organisation’ (127 references). The number of references made per code were disaggregated by district, to demonstrate inter-district variations in the comments made (see Additional file 1).

Based on the qualitative findings, each domain was classified as ‘simple’, ‘complicated’ or ‘complex’, in accordance with the Framework (see Additional file 2). Two domains—‘The Technology’ and ‘The Organisation’—were classified as ‘complex’, posing a substantial threat to adoption. The technology was considered to be complex because of reported charging issues (including battery faults), concerns about ongoing procurement, and potential to doubt or misinterpret results if inadequately trained. The organisation was considered to be complex because of infighting, high staff turnover, and the extensive work involved in implementation. These domains are also interdependent, for example, the need for ongoing training increases the work involved in implementation. Providing the device, and training staff in its use, is therefore not in itself enough to ensure success.

Several factors stood out as being more prevalent in districts with lower adoption: power struggles (mainly in District A), a lack of trust in the MoHS (only in District B) and mistrust in device accuracy (both A and B). Each reference included under the NASSS domains was subsequently coded as either a barrier or facilitator to adoption. The proportion of references coded as barriers was greater in districts with low adoption than those with high adoption (46%, 40%, 31% and 33% for Districts A, B, H and D, respectively). This may reflect more barriers faced, or a different mentality towards challenges.

#### The condition

Several participants commented that pregnant women delay coming to government health facilities, often first seeking advice or traditional medicine elsewhere—also reported by pregnant women in other studies [21]. This may contribute to women presenting with complex, urgent needs.

*The woman had delivered 5 days and she was brought to clinic, they said she saw devil […] When they came with that woman at the facility, when they came with her at the facility she was convulsing, and they had put garlic and others over her.—*District A

However, participants reported that CRADLE helps mitigate against late presentation by supporting prompt action.

*“The problem we were having, the pregnant women they delay to come to the health facilities because they need to seek advice from their, from their in-laws or from their elderly people in the villages. […] But with the help of the CRADLE machine, when she comes, immediately you will be able to monitor and detect whatever problem.*—District B

#### The technology

Participants across all districts reported that CRADLE was easy to use and improved the accuracy of vital signs recording.

*Because at first, they used to just give the, the vital signs that they feel like giving, but because of the CRADLE they now give us correct vital signs.—*District H

Incorrect use (e.g. poor positioning) could raise doubts about its accuracy. However, this is eased with ongoing support. Doubts about accuracy were more common in districts with low adoption (A and B).

*[Imitating staff members]: ‘Sister, that machine, that machine is difficult, when we use it, we don’t have the correct reading’ […] They do not, the thing would not read, it would go error, each time they take it, it go error, X. So that’s why they don’t like it, but they don’t know how to position the patient.*—District A

Interpreting the results (particularly the arrows) was also reported as challenging for some staff.

*Participant 1: Sister, the landmarks of the, the BP machine, the, the CRADLE is not actually difficult to use. It is the interpretation of the, the CRADLE that is where the problem is, especially with the arrow*

*Participant 2: Yeah*

*Participant 3: Arrow up, arrow down.*—District B

It was widely felt that one-off training was insufficient. Champions were not given funds to travel for in-person follow-up, so many devised alternative strategies to check on trainees.

*We do online teaching because we are far apart, the distance. So sometimes we have a [WhatsApp] group, so we go on, we will go online teaching.*—District H

Challenges with charging the device were frequently mentioned, including the battery not retaining charge, lack of power supply to facilities and national power cuts. A clear charging schedule was reported to help, but the task remained burdensome.

*Whenever someone is coming for that in-charges meeting, I give to the person, ‘please go with this machine and charge it, then when you are coming come back with it’. That’s how we are managing to charge them, though it is very difficult.*—District B

Concerns were also raised about the ongoing procurement of spare devices and parts (unavailable in SL). Participants worried about becoming reliant on a device that may subsequently be withdrawn. These concerns were mentioned more frequently in low-adoption districts.

*Like this, good things are brought in this country, so my worry is, in case we are used to this one and then this thing phase out, then there is nowhere we can get this machine, how are we going to deal again with our pregnant women? So this was my worry, that came in to my mind.—*District A

#### The value proposition

Champions in all districts unanimously reported that the device was desirable and effective. In keeping with the previous multi-site evaluation [12], the device was reported to support problem identification and management and met an urgent need for blood pressure machines. A novel finding is that Champions reported noticing a reduction in maternal deaths since using the device, with several providing personal accounts of this.

*Participant: We check the pressure, because of that arrow up and that light, that what prompt them to call me. They know that something is wrong because of that arrow […] They said sister we have checked her but the arrow is up and the red light is just bleeping, yes. […] So what I did, we give the loading dose for the Mag Sulf, then I insert Nifedipine under the tongue. I called for ambulance and they bring her to [hospital]. Yes, she was there for 2 weeks.*

*Moderator: Then? She survived?*

*Participant: Yes, she survived.—*District A

#### The adopter system

Participants were grateful to have been selected as ‘Champions’ and expressed a sense of pride and responsibility in the role.

*It was amazing for me to be selected among thousands of nurses. I feel honoured. It was indeed a feather to my cap at that time. I felt so proud. You know, even my working changed because I was now, I was chosen as a Champion.—*District H

Staff trained by Champions were reported to be grateful for the device and keen to learn new skills.  However, they often expected additional payment to attend training, even though training was conducted within their routine working hours. Many Champions felt this could be managed with transparent communication and encouragement.

*Participant 1: Here for us in Sierra Leone [laughs], at any time we think of money, anywhere you want to go as long as they’ve told you, ‘this is a training’, we expect money. […] I will just tap the back, ‘Today Sister, noto money business o, mi padi. Yu no na fo le wi do sum ting, let d work go befo, yu kno say wi try for le all cam fo one’. (Today Sister this is not a money thing, my friend. You know it is for us to do something, let the work come first, you know we are all in this together) […]*

*Participant 2: Yes, the asking for money, it was at the beginning! [other participant laughs] But for now they have already got the knowledge, everybody is eager.—*District H

Champions reported that pregnant women appreciated having their vital signs taken and the results communicated to them, and this led to increased facility attendance. This supports earlier reports that women at sites across Africa and Asia (n = 41) unanimously liked the device [10].

*The Matron told me that er, they are now getting an inflow, influx of patients, more than before. You know patients are going out, telling people ‘eh, go now to [names centre] they have got a new device that can help to detect whatever problem you have’.*—District B

#### The organisation

Participants identified several barriers to implementation at facility level. Firstly, it was hard for Champions to access facilities, particularly during raining season, and the associated cost often exceeded the amount of money they had for travel. Many reported making sacrifices to overcome these barriers because they felt compelled to supply the device to others.

*I had to take bike for the rest of the day [unintelligible], but it’s because of I like the job and that makes me Champion, because I have to sacrifice. I have to give more so that the next facility will gain then they will have their CRADLEs. If you look, look for the amount, it’s too small. But we sacrifice so that the job will go well. So that’s why we spent a lot of money.—*District D

High staff turnover within the MoHS was also identified as a challenge, as Champions were often the ones training new staff. Follow up and encouragement from the District Health Management Team (DHMT) and implementing organisation was reported to motivate Champions to persist in spite of these challenges.

*She was motivating us to continue with the training and it is very good because to be frank when somebody is there to say, ‘continue doing this, continue doing this’, you will hold on to it the more and you will continue to do it.*—District H

At facility level, power struggles between Champions and trainees were commonly reported, particularly where normal hierarchy was challenged. Internal professional conflicts could also deter staff from attending training, even when the conflict did not directly involve the Champion. These issues were mentioned by all districts but primarily by District A (lowest adoption).

*Participant: There is this fight between the CHOs (Community Health Officers) and the... [utterances from group: ‘midwives’], and the Midwives [utterances from the group: ‘yes, yes], they used to fight. So, in the CHCs, you will have nurses in favour of the CHO, they will be with the CHO, and you will have nurses in favour of the maternity unit. So that fight is there.*

*Moderator: In-fighting?*

*Participant: Yes, in-fighting [utterances from group: ‘in-fighting’]. So if you the midwife take like this cradle, those that are for the CHO, will not participate. Only those that are in the maternity will participate.*—District A

Some participants felt that conflict could be avoided through transparent and sensitive communication with staff prior to training, particularly with the facility in-charges.

*They will say Sister, Sister [name] you are a leader. I say ‘why?’ They say, ‘we did all sort of things to you when you came here but you never got annoyed’. I said, ‘why should I got annoyed? I met you here, you know the place, so if I got annoyed, I would not know the centre. I rely on you, so that is why. You are my bosses, I met you here’. They will laugh. So now all of them have interest.*—District A

A few reported developing a stronger bond with other staff members and improved multi-disciplinary working as a result of the programme.

*“It’s important because without being Champion we are not interaction. When you came, you sit by cadre. I’m an SECHN* (State Enrolled Community Health Nurse)*; I go close to the SECHN instead of being close to the MCH Aid. But through the CRADLE makes me to interact with them.—*District D

#### The wider system

Few comments were made relating to the professional, sociocultural, and legislative context. However, those made were supportive of adoption. In particular, engaging community leaders supported implementation and promoted community buy-in.

*I called the community leaders, stakeholders and tell them about the great thing that has happened to us and I told them the functions of this CRADLE. They said, ‘you know what, we are going to set aside a day to name this CRADLE’. You know those people, so, so people they like to, they like merriment. They went and call* all *[emphasis added] the stakeholders from the catchment areas, call a very large meeting. They entertain us, they cook—bring and share, it was bring and share. The villagers will come with their own and we cooked, we eat ...* —District H

*I went there, explained to him (Paramount Chief) about the importance of this machines and he welcomed the idea and he was very happy. And he told the driver, his driver fuelled his car, and the driver was taking me around the facilities to go and do my trainings.*—District B

#### Embedding and adaptation over time

A few Champions doubted that the MoHS could sustain the use of CRADLE independently and several commented that continuation of external support was needed (predominantly District B—low adoption).

*Because we know the government of, our government, sometimes something will come out very, very good. It will start good, like very speed, it will just die out slowly, slowly. We pray that this will not happen to CRADLE machine.*—District B

*We have similar things like for the drugs […] You will requisite, at the end of the day they will tell you the drug is not available […] continue to report, continue to report and there is no action, this is our fear.—*District B

However, more Champions, across all districts, felt they could take ownership of sustainability efforts—integrating with existing activities and advocating to the MoHS.

*We need to come together and form a very strong pillar […] if we come out with our voices telling them the importance of this machine, telling them how we need more of this machine, they will in turn go to the central government.* – District B

*Most times supervisions come, if you are in those provinces, these vehicles come, go off for supervision. If they are going there you just use that opportunity to go with them, go to the facility, then you do your recap.*—District A

Contrasting opinions in District B were reflected by how Champions responded to new challenges, for example, staff turnover.

*Participant 1: I have suggested that they train another Champion*

*Moderator: Who is the they? Who is the they?*

*Participant 1: The DHO (District Health Officer) or support from you people [the implementing organisation]*

*[…]*

*Participant 2 [addressing Champion 1]: You are already trained; you have already trained them so it’s better that you select somebody among those that you have trained […] I think it’s better that way—we are doing it for ourselves—*District B

## Discussion

### Adoption of CRADLE in SL

Based on quantitative adoption indicators, average adoption levels in this study were lower than seen in Freetown during the CRCT (77.5% versus 96.2%). This likely reflects differences in the adoption indicator used: ‘percentage of facilities trained’ in this study versus ‘percentage of clinical areas using solely the CRADLE at 12 months’ in the CRCT. The former reflects whether Champions offered training and the facility accepted it (i.e. *engagement* with the intervention), whilst the latter measures *use* of the innovation *post*-training. The difference in adoption levels may therefore reflect that whilst staff are willing and able to adopt the device once adequately trained and supported, barriers to implementation during scale-up may hinder engagement at the outset. Furthermore, adoption was not uniform across all districts, with some districts reaching adoption levels of 90%. Exploring this variation has helped identify factors that may contribute to non-adoption and potential strategies to overcome them.

### Complexity impacting upon adoption

In the previous evaluation of the CRADLE CRCT, few sites mentioned barriers to adoption, primarily citing the device’s sensitivity to movement [12]. In contrast, this study identified many barriers, with substantial complexity identified around the technology and the organisation. Potential strategies to manage these are discussed, but it is important to note that complexity cannot be completely solved and may even increase with time [22]. Periodic monitoring throughout the scale-up in SL is therefore recommended to help manage emerging complexities. This may be supported by practical, purpose-designed tools, such as the NASSS-CAT Tools [23].

#### Organisational factors

Several barriers to implementation at facility level were identified by Champions. The district with lowest levels of adoption mentioned power struggles and infighting frequently, and far more than other districts. Intra- and inter-professional conflicts in healthcare settings have been reported elsewhere in Sub-Saharan Africa in relation to staff burnout [24], but not previously in relation to implementation of health programmes. This study suggests that professional relationships may influence health technology adoption in Sierra Leone, and so should be considered when designing, implementing, and evaluating new programmes in this context. To help avoid professional conflicts, Champions reported that initial consultation with facilities (e.g. about the programme, the plan for training, and the lack of financial incentives) must be communicated transparently and carefully. To respect existing hierarchies, this information may be best relayed by someone in a position of authority (e.g. a member of the DHMT), and/or Champion selection should consider candidate’s inter-personal skills.

Some Champions independently took the initiative to engage community leaders in their area. These Champions reported that the community leaders assisted them with implementation and enabled them to gain the support of other healthcare providers and the wider community. Involving Community Leaders in Public Health interventions is widely encouraged [25] and was critical in controlling the 2013 Ebola outbreak in SL [26]. This strategy could therefore be actively promoted to CRADLE Champions in ongoing scale-up efforts.

#### Technological factors

In keeping with earlier CRADLE studies [10, 12, 27], respondents generally found the device easy to use, but small errors in application (e.g. incorrect positioning) could lead to staff mistrust. A novel finding was that interpretation of the arrows (critical for guiding clinical management), was difficult for some staff. Modifying this aspect of the training package—co-creating new materials with staff who found it challenging—may help to ensure effectiveness [28]. Champions also reported using their own strategies to support training of staff (e.g. WhatsApp). Rather than ensuring implementation fidelity, these sorts of initiatives and contextually appropriate modifications should continue to be encouraged throughout scale-up to promote adoption [29].

Difficulty charging the device was mentioned frequently and could lead to abandonment if not addressed. Approximately 40% of maternal and child health posts in Sierra Leone report having no electricity source [30], and previous studies have commented that unstable power supplies can limit technological adoption in Sub-Saharan Africa [31]. CRADLE Champions found novel ways to manage this issue in the short term, such as charging devices outside of the health facility. Further, ‘Solar suitcases’—easy-to-use solar electric systems that can power small medical devices—are being rolled out in remote maternal health facilities nationally and may help mitigate against this issue to an extent [32]. However, in the long term, providing a stable power supply to all health facilities is a clear priority. Champions also reported faults with the battery itself (e.g., not retaining charge), which were similarly reported in the CRCT [11]. The mechanisms of these faults are yet unknown and Medical Equipment Technicians (Ministry of Health and Sanitation staff who have been familiarised with the device) are now undertaking further investigation into the issue. In the interim, it will be important to ensure that training on device maintenance, including correct charging practices, remain part of the CRADLE training package.

Finally, concerns were also raised about the MoHS’ capacity to sustain the programme, particularly in relation to procurement of devices and parts. This may reflect fragmentation in the healthcare system and disparity between those holding decision-making power and resources (central government, external donors) and those responsible for delivering services at district level [15]. Several steps were taken during implementation to promote sustainable training and procurement, including adding the device to the National Health Facility Equipment List, and incorporating CRADLE within the University obstetric curricula and the National Emergency Obstetric and Newborn Care (EmONC) training programme. Informing Champions and District Management about such actions, and keeping them informed regarding ongoing procurement, may help to allay sustainability concerns.

### Transferability of the NASSS framework

Empirical work to develop NASSS and subsequent applications has been conducted entirely in high-income settings [14, 33]; this study was the first to test its utility in a low-income setting. All qualitative data codes in this study aligned with the NASSS domains, suggesting the framework translates to low-income contexts. However, further application in other low-income contexts should be conducted to support or refute this. As professional conflict was identified as a threat to adoption in this context, it may be valuable to add questions about this to the NASSS guidance.

### Limitations

FGDs were held in only four districts; completing focus groups in other districts may have yielded different results. However, when coding data, no new codes emerged after analysis of the third FGD, suggesting data saturation was reached. The use of FGDs may have increased the risk of social desirability bias. However, Champions reported many barriers, and conflicting opinions were offered within the same FGDs, suggesting participants felt able to speak openly. The evaluation is specific to the adoption of CRADLE intervention and SL, limiting the generalisability of findings. However, contextual information has been provided to allow readers to draw from the findings where appropriate. The author was the Programmes Manager of the implementing organisation of CRADLE during this phase of scale-up. There are potential benefits of this ‘embedded implementation research’ approach, including better contextual understanding of the findings [34]. However, this also increased the risk of investigator bias. To mitigate this, questions were designed to be non-leading, and the analysis reflective of the data. The author is also White British, which in light of the colonial history of SL may have created a power imbalance limiting participants’ willingness or ability to voice their opinions [35]. To mitigate this, the lead moderator of FGDs was a Sierra-Leonean Midwife, known to the Champions.

## Conclusion

This study found considerable inter-district variation in adoption of the CRADLE device in SL. Complexity was identified in two NASSS domains, ‘the technology’ and ‘the organisation’ with barriers to adoption including infighting, high staff turnover, charging issues, and concerns about ongoing procurement. Device provision and training are therefore unlikely to be enough to ensure its sustained adoption and long-term impact. Strategies identified to mitigate against issues and promote sustained adoption in SL include: early, transparent communication to facilities about training plan (e.g. lack of financial incentives, rationale for breaking hierarchies where applicable); selecting Champions with strong soft-skills (particularly conflict resolution, problem solving and tenacity); encouraging localised adaptations during implementation (including involvement of relevant community leaders); further investigation into and resolution of device charging issues; and informing all cadres of staff of updates regarding ongoing device and parts procurement. This study has implications for the ongoing scale-up of the CRADLE in SL and for those implementing or evaluating similar technology innovations in the region.

## Supplementary Information

**Additional file 1.** For a table of the coding list arranged by NASSS domains.

**Additional file 2.** For a tabulated summary of the findings, and given classification, for each domain.

## Data Availability

The data supporting the conclusions of this article are included within the article and its additional files.

## Abbreviations

CRADLE: Community blood pressure monitoring in Rural Africa: Detection of underLying pre-Eclampsia
NASSS: Non-adoption, abandonment, scale-up, spread, sustainability
MMR: Maternal mortality ratio
CRCT: Cluster-randomised controlled trial
MoHS: Ministry of Health and Sanitation
FGD(s): Focus Group Discussion(s)
DHMT: District Health Management Team
SECHN: State Enrolled Community Health Nurse
