# Supplementary material for: Scale-up of a novel vital signs alert device to improve maternity care in Sierra Leone: a mixed methods evaluation of adoption
Source: Reprod Health. 2023 Jan 6;20:6. doi: 10.1186/s12978-022-01551-2 (PMC9817393; doi:10.1186/s12978-022-01551-2)
Supplement: Supplementary file 1 — Additional file 1. For a table of the coding list arranged by NASSS domains. [file 12978_2022_1551_MOESM1_ESM.docx]

Additional file 1 - *Coding list arranged by NASSS domains*

|  | **Refs. per district** | | | | **Total** |
| --- | --- | --- | --- | --- | --- |
|  | **A** | **B** | **H** | **D** |  |
| **1. The Condition** | | | | | **5** |
| 1.1 Pregnant women delay seeking healthcare | 2 | 2 | 0 | 1 | 5 |
| **2. The Technology** | | | | | **152** |
| 2.1 Accuracy of the device |  |  |  |  |  |
| 2.1.1 Improves accuracy of, and belief in, vital signs monitoring | 6 | 5 | 6 | 1 | 18 |
| 2.1.2. Some doubt accuracy - supported by follow up and experience | 8 | 6 | 2 | 1 | 17 |
| 2.3 Knowledge and support required to use the technology | | | | | |
| 2.3.1 Ease of use and interpretation | | | | | |
| 2.3.1.1 Strategies used to promote understanding | 0 | 7 | 1 | 0 | 8 |
| 2.3.1.2 Easy to use | 1 | 4 | 1 | 0 | 6 |
| 2.3.1.3 Difficulty interpreting results - especially arrows | 0 | 2 | 1 | 0 | 3 |
| 2.3.2 Follow up post-training | | | | | |
| 2.3.2.1 Follow up by Champions | 13 | 6 | 8 | 4 | 31 |
| 2.3.2.2 Internally led follow up within facilities | 5 | 2 | 8 | 1 | 16 |
| 2.2 Charging | | | | | |
| 2.2.1 Charging problems | 1 | 3 | 4 | 7 | 15 |
| 2.2.2 Solutions to charging problems | 1 | 4 | 4 | 1 | 10 |
| 2.4 Maintenance |  |  |  |  |  |
| 2.4.1 Concerns about long-term supply of devices and parts | 4 | 6 | 3 | 1 | 14 |
| 2.4.2 Methods used to maintain the device | 1 | 1 | 7 | 1 | 10 |
| 2.4.3 Device faults and poor handling | 3 | 1 | 0 | 0 | 4 |
| **3. The Value Proposition** | | | | | **70** |
| 3.1 Improves clinical management - reducing maternal death | 10 | 14 | 7 | 9 | 40 |
| 3.2 Timely - Lack of other BP machines | 1 | 6 | 3 | 1 | 11 |
| 3.3 Makes work easier | 2 | 5 | 1 | 2 | 10 |
| 3.4 Helps identify problems the woman does not communicate | 1 | 2 | 0 | 0 | 4 |
| 3.5 Belief that device is special or magical | 1 | 2 | 0 | 0 | 3 |
| 3.6 Barriers to acting on device output | 1 | 1 | 0 | 0 | 2 |
| 3.7 Needs to be used alongside clinical reasoning | 0 | 0 | 0 | 1 | 1 |
| **4. The Adopter System** | | | | | **105** |
| 4.1 CRADLE Champions (implementors) – roles, practices and identities | | | | | |
| 4.1.1 Feeling of pride, gratitude and responsibility to be a 'Champion' | 9 | 13 | 9 | 9 | 40 |
| 4.1.4 Low confidence in ability to teach - eased through experience | 0 | 0 | 0 | 6 | 6 |
| 4.1.5 Request to have more CRADLE Champions | 0 | 0 | 1 | 3 | 4 |
| 4.2 Healthcare providers (end users) – roles, practices and identities | | | | | |
| 4.2.2 Staff grateful for support and keen to learn | 0 | 8 | 7 | 6 | 21 |
| 4.2.1 Staff expect incentives to attend training | 5 | 4 | 7 | 0 | 16 |
| 4.2.2 Increases HCP commitment to helping women | 1 | 2 | 1 | 0 | 4 |
| 4.3 Women (patients) like the device, increasing facility attendance | 5 | 2 | 3 | 0 | 10 |
| **5. The Organisation** | | | | | **127** |
| 5.1 Changes to team interactions and routines | | | | | |
| 5.1.3 Power struggles and in-fighting | 21 | 1 | 2 | 3 | 27 |
| 5.1.1 Improves staff relationships and MDT communication | 0 | 4 | 7 | 3 | 14 |
| 5.1.2 Differences in training own staff versus others | 0 | 0 | 0 | 3 | 3 |
| 5.2 Work involved in implementation | | | | | |
| 5.2.1 Logistical and financial barriers – requires personal sacrifice | 1 | 9 | 4 | 13 | 27 |
| 5.2.2 Requires transparent communication and mediation skills | 11 | 0 | 7 | 6 | 24 |
| 5.2.3 Follow up of CRADLE Champions helpful | 1 | 4 | 9 | 7 | 21 |
| 5.2.4 High staff turnover - ongoing training required | 2 | 2 | 2 | 1 | 7 |
| **6. The Wider System** | | | | | **9** |
| 6.1 Integrating with Training Institutions | 0 | 0 | 2 | 1 | 3 |
| 6.2 New law about follow up of maternal deaths | 0 | 1 | 1 | 0 | 2 |
| 6.3 Rollout supported by community leaders | 0 | 1 | 3 | 0 | 4 |
| **7. Embedding and Adaptation over time** | | | | | **26** |
| 7.1 Belief in own capacity to sustain and integrate CRADLE within MoHS | 1 | 7 | 2 | 5 | 15 |
| 7.2 Requesting for support from external partners | 0 | 5 | 3 | 1 | 8 |
| 7.3 Doubt about MoHS capacity to sustain the use of CRADLE | 0 | 3 | 0 | 0 | 3 |
