# Supplementary material for: Scale-up of a novel vital signs alert device to improve maternity care in Sierra Leone: a mixed methods evaluation of adoption
Source: Reprod Health. 2023 Jan 6;20:6. doi: 10.1186/s12978-022-01551-2 (PMC9817393; doi:10.1186/s12978-022-01551-2)
Supplement: Supplementary file 2 — Additional file 2. For a tabulated summary of the findings, and given classification, for each domain. [file 12978_2022_1551_MOESM2_ESM.docx]

Additional file 2 – Classification of each domain according to main findings and NASSS guidance

| **Domain** | **Summary of findings from FGDs** | **Classification** |
| --- | --- | --- |
| **The condition or illness** | - Patient may present with more complications as a result of late presentation/seeking other advice/treatments first | Complicated |
| **The technology** | - Directly and transparently measures changes in the condition  - Requires ongoing training (knowledge generated contested as a result of incorrect use)  - Difficulties charging the device  - Unable to procure spare parts or devices in country | **Complex** |
| **The value proposition** | - The device is highly desirable to staff and patients  - Reported to impact on quality of care provision and reduce maternal mortality/morbidity | Simple |
| **The adopter system** | - Staff happy to receive the device and only positive comments made in relation to changes in professional identity  - Device accepted by women and their support network (no new tasks expected of them. Some communities voluntarily offered support)  - Staff can expect incentives to attend training | Complicated |
| **The organisation** | - Power struggles between Champions and trainees and in-fighting within facilities (reported to be present prior to and independent of the CRADLE intervention) could be a barrier to innovation  - Extensive work involved in implementation, requiring ongoing commitment and at times personal sacrifice from Champions | **Complex** |
| **The wider system** | - Midwifery schools and MoHS supportive  - New law increasing focus on prevention of maternal deaths  - Community leaders supportive | Simple |
| **Embedding and adaptation over time** | - Concerns about ability to maintain the programme without external partner support  - Many Champions proactive and independent in approach to overcoming challenges | Complicated |
